# Blockchain-based secure MEC model for VANETs using hybrid networks

**DOI:** 10.1038/s41598-025-27682-7

**Published:** 2025-12-16

**Authors:** G. Vijay Goud, Rajesh Arunachalam, Surendra Kumar Shukla, K. Saranya, Sumanth Venugopal, Preethi Palanisamy

**Affiliations:** 1 Department of Electronics and Communication Engineering, Saveetha School of Engineering, Saveetha Institute of Medical and Technical Sciences, Thandalam, Chennai, Tamilnadu 602105 India; 2 Department of Computer Science and Engineering, Amity school of Engineering and Technology, Amity University, Noida, Uttar Pradesh 201303 India; 3 Department of Computer Science and Engineering, Koneru Lakshmaiah Education Foundation, Vaddeswaram, Guntur, Andhra Pradesh 522302 India; 4 Manipal Institute of Technology Bengaluru, Manipal Academy of Higher Education, Manipal, India; 5 Centre for Research Impact & Outcome, Chitkara University Institute of Engineering and Technology, Chitkara University, Rajpura, Punjab 140401 India

**Keywords:** Vehicular ad-hoc networks, Quality of service, Secured multi-access edge computing, Adaptive and dilated hybrid network, Residual long short-term memory with gated recurrent unit, Random number updated skill optimization algorithm, Homomorphic with elliptic curve cryptography, Energy science and technology, Engineering, Mathematics and computing

## Abstract

Vehicular Ad-hoc Networks (VANETs) are a type of mobile ad-hoc network that enables vehicles to interact with one another and roadside infrastructure. Multi-Access Edge Computing (MEC) provides a promising solution by positioning storage and computation resources closer to the network edge. This helps to reduce the latency and improve performance. The combination of MEC and blockchain enhances data processing and security. This integration improves privacy safeguards, prevents fraud, and supports trusted communication within VANETs. Consequently, this proposed model aims to develop an innovative approach that leverages these technologies. The main objective of the implemented technique is to create a blockchain architecture powered by deep learning, which ensures the safety of VANETs. The network architecture consists of three layers: perception, edge computing, and services. The main goal of the initial layer is to protect the privacy of VANET data through blockchain activities. The perception layer processes data using edge computing and cloud services. The service layer ensures data protection by through the blockchain technology and storing information in a public cloud. The last layer focuses on addressing user demands for throughput and Quality of Service (QoS). The proposed framework is good for assessing the dependability of vehicle nodes stored on the blockchain. To accomplish node authentication, an Adaptive and Dilated Hybrid Network (ADHyNet) is used. In this approach, the Residual Long Short-Term Memory (Res-LSTM) with Gated Recurrent Unit (GRU) forms the ADHyNet, where the Random Number Updated Skill Optimization Algorithm (RNU-SOA) is used to optimize the hyperparameters. Finally, the encryption process is carried out using Homomorphic Encryption combined with Elliptic Curve Cryptography (HECC) to secure data. This process ensures that confidential user information is protected against unauthorized access. The functionality of the system is thoroughly assessed and simulated. The suggested technique outperforms well than other approaches in terms of data security in VANET.

## Introduction

MEC is a software-defined framework designed to deliver the storage and computation capabilities in the network. This architecture enables low-latency services but relies on an implicit trust model among its components, introducing potential vulnerabilities that attract various threats^1^. However, blockchain technology offers distinct features that enhance security in MEC-based networks^2^. Security is especially crucial due to the inherent vulnerabilities in the VANET environment. It involves defending the network against threats such as unauthorized access, message tampering, and violations of user privacy. Recent studies show that device-enhanced MEC is affected by factors like connectivity and resource availability, which can process resource-intensive tasks quickly. Despite this, MEC devices face significant challenges regarding data integrity, security, and privacy^3^. When application data and tasks are shared across devices owned by different users, several concerns arise to maintain the integrity of applications^4^. This is primarily due to the difficulties arising while verifying the trustworthiness of all participating nodes. As a result, malicious nodes may attempt to undermine the system’s integrity^5^. Blockchain technology addresses these concerns by operating on a distributed ledger that functions without a central authority. This decentralized structure reduces single points of failure and increases resilience to attacks. It allows transactions to be securely recorded and updated. Once a transaction is added, it cannot be modified in order to ensure data integrity. This guarantees that data transferred between vehicles and infrastructure remains consistent, accurate, and reliable^6^. Additionally, blockchain can securely manage identities and access permissions, which is essential for multiple devices owned by different users^7^.

Recent research in the VANET domain has focused on various aspects of information security, such as maintaining data integrity, validating node identities, and preventing cyber attacks^8^. These are crucial for safe data processing and communication^9^. In centralized systems, attackers can exploit to identity theft, data tampering, or network disruption^10^. Moreover, the deep learning techniques are adapted in power recommendation systems, which analyze data and extract meaningful insights^11^. These techniques help improve efficiency, safety, and traffic management. For example, on-board units equipped with deep learning models can detect abnormal traffic patterns in real-world scenarios. However, most deep learning solutions still rely heavily on centralized servers, which limit scalability. As the network grows, computational demands increase and delays become more frequent^12^. Emerging solutions aim to address this by enabling collaborative learning without transmitting sensitive data to centralized servers. This approach improves both security and performance as the network scales.

Implementing secure node authentication in VANET, deep learning-based models can authorize vehicles in the network^13^. This significantly reduces the risk of unauthorized access and malicious behaviour^14^. The integration of blockchain establishes a decentralized ledger where all data transactions are permanently recorded^15^. Once the data are stored, they cannot be altered or removed. Combining deep learning with MEC enables the system to scale efficiently as the network expands^16^. These models can handle the vast amount of data generated by numerous vehicles. Simultaneously, the decentralized nature of blockchain accommodates an increasing volume of transactions without degrading performance^17^. Moreover, deep learning models, trained on extensive datasets, can detect patterns and make predictions, enhancing decision-making in areas such as traffic control and emergency response.

The main contributions of the proposed work are listed as follows:

- A secure MEC model for VANET is developed to enable efficient data communication between vehicles and edge servers. It incorporates multiple security layers, including blockchain-based node authentication and data encryption, to protect the network against various threats. This layered approach is crucial for minimizing unauthorized access and reducing potential risks, thereby ensuring network integrity. By utilizing blockchain technology, the proposed model creates a decentralized ledger that permanently records all data transactions. A secure MEC architecture in VANETs offers several advantages. These include enhanced reliability, improved security, lower latency, and greater scalability. This approach effectively reduces the likelihood of data breaches. It also accelerates the processing of safety–critical information and supports efficient management of the dynamic and rapidly evolving VANET environment.
- The objective is to develop a blockchain mechanism integrated with node authentication and data encryption in VANET. This integration aims to achieve greater resilience against cyber threats. It enhances data security, supports secure resource management, and builds trust among network entities. Sensor data and traffic events exchanged between vehicles and Road Side Units (RSUs) are stored as blockchain transactions. This creates a verifiable and tamper-proof record, which ensures the authenticity of the shared information.
- ADHyNet is a specialized neural network architecture designed for node authentication in VANET environments. The ADHyNet-based mechanism ensures security and trust. It helps prevent malicious vehicles from joining the network. This mechanism also verifies the integrity of transmitted messages. The ADHyNet network integrates Res-LSTM and GRU architectures. This combination facilitates gradient flow during training and addresses the vanishing gradient problem. These properties make it highly suitable for processing sequential data, significantly improving the efficiency of node authentication.
- The HECC model is developed for secure data encryption in VANETs. Its main goal is to enable secure communication between vehicles and RSUs. This encryption technique ensures the safe exchange of information. Elliptic Curve Cryptography (ECC) provides strong security using relatively smaller key sizes compared to other cryptographic methods. As a result, it offers faster encryption and decryption operations. This efficiency is especially important in resource-constrained environments like vehicular networks.
- An RNU-SOA strategy is also developed to enhance the performance of ADHyNet-based authentication. It fine-tunes hyperparameters that affect the model’s learning capability. The algorithm uses skill optimization combined with random number enhancement to search for optimal network parameters. This improves node authentication performance in VANETs. The inclusion of random number updates in traditional skill optimization leads to better convergence and more efficient parameter tuning, particularly in complex scenarios.

The overall organization of this framework is given in the points below. Reviews of the proposed models are described in Section "Literature survey". The structural descriptions of the proposed secured MEC model in VANET are provided in Section "Implemented secured MEC model in VANET using blockchain-assisted deep learning: structural description". The descriptions of the deep learning-based trust node authentication process are provided in Section "Trusted node authentication using dilated hybrid deep learning framework". Detailed information on encryption algorithms is elucidated in Section "Blockchain-based secure data storage using encryption algorithms in MEC". The results and discussions are given in Section "Results and discussion", and a summary of the work is presented in Section "Conclusion".

## Literature survey

This section incorporates various existing literature works in the domain of VANET security and authentication. The problems involved in the prior approaches and how those issues were solved in this research work are elaborated in this section.

### Related works

In 2025, Kalidoss et al.^18^ proposed a blockchain-based deep learning technique to enhance VANET security. The model consists of three main layers: edge computing, perception, and service layers. The blockchain mechanism was used in the initial layer to maintain the privacy of VANET data. The perception layer combines edge computing with cloud-based services. The final layer addresses user requirements related to throughput and QoS. Node authentication was carried out using the Adaptive Dilated (AD)-GRU model, with hyperparameter optimization improving authentication performance. Simulation results confirmed that the developed system provided superior security in the VANET environment.

In 2023, Zhang et al.^19^ introduced a multi-layered grid-based spatial framework to detect terrestrial adversaries. This model effectively detected the attack-defense interaction of laser wireless power transfer in aerial computing settings. Game theory was applied to the model to optimize resource distribution related to laser wireless power transfer. The authors derived the mixed-strategy Nash equilibrium for both asymmetric and symmetric tile grid scenarios. Simulation results showed that this framework significantly outperformed traditional models in terms of privacy protection.

In 2023, Asad et al.^20^ proposed a secure and efficient blockchain-based federated learning strategy to enhance communication proficiency and data privacy within VANETs. The federated learning model divided computation tasks between the base station and vehicles. The model integrates blockchain with federated learning to ensure secure data transfer between RSUs, vehicles, and the cloud server. Simulation results confirmed that the accuracy of detecting membership inference attacks was improved in this model.

In 2023, Sarah et al.^21^ proposed an MEC technique to enable Ultra-Reliable Low Latency Communications (URLLC) services by processing and storing data closer to user equipment. Data processing occurs at the edge network, with storage in cloud systems. The resource optimization issue in MEC was defined based on communication requirements, resulting in lower energy usage for communication.

In 2022, Le et al.^13^ focused on a trust module for enhancing reliability in MEC node interactions during service migration tasks. MEC nodes used a calculated trust score to make migration decisions for services and user information. The proposed technique used the dynamic distance algorithm and EigenTrust for trust evaluation. Simulation results showed that this approach performed better in various scenarios and communication ranges.

In 2023, Zhang et al.^22^ introduced a novel architecture for node authenti**cation,** integrating Digital Twin technology with MEC. The framework also introduced an efficient method for building a knowledge-sharing machine tool swarm. The model incorporated three key methodologies: knowledge-based cloud learning, digital twin-based machine tool swarm, and MEC-assisted model deployment. The prototype and application examples confirm the feasibility of the developed framework.

In 2023, Ali et al.^23^ introduced a three**-**layer MEC model that integrates a trust-sensitive load-balancing decision-making mechanism. A Q-learning algorithm was utilized to evaluate trust values, and task trust. Then, indirect trust relationships were recorded through a blockchain technology. Blockchain ensured secure and transparent resource management, enhancing the security and reliability of the system.

In 2022, Gyamf et al.^24^ proposed an adaptive Machine Learning-Based Security (MLS) task offloading framework to ensure secure and reliable connectivity between MEC servers and edge devices. The MLS task offloading and synchronization challenges were formulated as mathematical models and solved using Markov transition probability and maximum likelihood estimation for clock offset. Simulations showed that the framework ensured robust security for the edge network while achieving low latency and high synchronization accuracy.

In 2024, Kumar et al.^37^ have proposed a secure-lightweight data collection and sharing framework for secure and smart transmission in the 6G network. Here, the data transmissions were secured using blockchain technology with a voting-based consensus technique. The developed scheme offered higher authentication and security to the Internet of Vehicles (IoV). In addition, they effectively reduced the communication and validation expense than the prior authentication mechanism.

In 2024, Kumar et al.^38^ has designed a secure monitoring system by considering the IoT and other computing devices. In this phase, the security was computed through informal and formal security analysis procedures. Finally, the validation outcomes displayed that the security of the developed network was better than the classical mechanism and also consumes minimal energy to transmit the information.

In 2024, Kumar and Ali^39^ have proposed a new smart contract-based authentication technique with blockchain technology to maintain network security. The developed mechanism was better in handling overall network transparency, privacy and security. Moreover, this technique effectively reduced the delay and accomplished superior outcomes.

In 2024, Kumar and Ali^40^ have developed a new authentication technique in the blockchain with a controlled access mechanism. The major objective of the developed scheme was to handle real-world information and also enhance privacy and security in different classes.

In 2025, Kumar and Ali^41^ have implemented a secure data sharing technique by considering the multi-factor authentication and smart contracts. This technique was good in managing the higher robustness and achieving lower latency while communication was performed. Analysis outcomes displayed this technique effectively maintained the scalability, security and computational overhead than the classical techniques.

### Problem statement

Data processing at the network edge, near the user, to ensure low latency is known as secured MEC. Similarly, a secured MEC for the VANET sector is a system that uses MEC concepts to offer security and reliability by deploying processing power at the network edge. This allows for quick data processing and integrates robust safety features to protect sensitive vehicle information. One of the primary challenges is managing vehicle identity and authentication. Moreover, VANETs are inherently open and short-lived, making resource allocation across edge nodes more difficult. Therefore, this work proposes a novel secured MEC model with blockchain for VANET, designed to ensure data integrity, privacy, and trust among participating vehicles without relying on a central authority.

- Traditional secured MEC models with blockchain for the VANET sector face significant challenges in effective resource allocation. They struggle to adapt to dynamic network conditions. To address these challenges, the deep learning technique is employed to optimize resource utilization and reduce latency. This technique improves resource allocation efficiency and enhances adaptability across various scenarios.
- Another issue with traditional models is data privacy and potential vulnerabilities in decentralized data storage. Additionally, they face difficulties in managing access control across multiple edge devices. To mitigate these concerns, hybrid encryption is implemented to secure data at various stages of processing and transmission. This enhances the overall security and privacy of the system, while also managing vulnerabilities in decentralized data storage. Furthermore, it provides robust access control across multiple edge devices.
- Traditional models also struggle with limited computational power at the edge and frequent changes in network topology due to vehicle mobility. To address these issues, residual connections can be employed to improve deep network training. This method reduces gradient problems, boosting computational power and allowing information to flow directly without requiring frequent changes to network topology.
- Another challenge faced by traditional models is limited scalability and bandwidth accessibility when handling large volumes of real-time data in a dynamic vehicular environment. This can significantly impact model accuracy. To solve these problems, the dilated convolution technique can be used to enhance prediction accuracy. It improves the scalability of the model and boosts bandwidth accessibility, enabling more accurate processing of large data volumes.

Advancements and disadvantages of the traditional approaches for secured MEC environments are given in Table 1, and the section that gives the research gaps.

**Table 1:** Advancements and disadvantages of the existing secured MEC with blockchain models for the VANET sector.

Author [citation]	Techniques	Features	Disadvantages
In 2025, Kalidoss et al*.*^18^	AD-GRU	It significantly improves data security and consistency for attaining better transmission	Processing data closer to the consumer may result in delay, and integrating blockchain may cause additional latency
In 2023, Zhang et al*.*^19^	Multi-tier tile grid-aided spatial structure	It efficiently offers aerial sites for laser charging	The model may have reduced complexity and scalability in managing the laser beam allocation across multiple devices
In 2023, Asad et al*.*^20^	SEBFL	It offers an extra layer of security for VANETs It can preserve the confidentiality and credibility of vehicles	As the network expands, scalability becomes an issue, potentially causing delays in blockchain verification procedures
In 2023, Sarah et al*.*^21^	MEC resource allocation	It facilitates improved context-awareness, security, and dependability It ultimately can reduce the total latency It efficiently helped to solve more complex problems	It may need a smarter integrated multi-paradigm resource orchestration The model may be limited by the intelligent resource allocation strategy
In 2022, Le et al*.*^13^	EigenTrust and RLIoT	It offers increased reliability in the context of setting up the service	One major challenge is due to live migration for specialized services like gaming or video streaming It may not analyze the node status
In 2023, Zhang et al*.*^22^	Knowledge-sharing intelligent machine tool swarm	It verifies the high fidelity of DTMT It efficiently improves the secure sharing of data across DTMTs, and the latency of the model is low	Real-time data processing, low latency, and distributed intelligence, with potential across industries are complex and difficult The model may have unstable response times and decreased efficiency
In 2023, Ali et al*.* ^23^	Three-layer MEC model	It generates improved application performance, load balancing process and produces reduced latency It enhances task load balancing with better security, which is offered using a zero-trust model	This method does not contribute multiple identity factors across MEC servers
In 2022, Gyamf *et.al* ^24^	ASTO	It effectively protects the network It generates reliable security in a distributed, large-scale IIoT network	This method may have limited intrusion detection capacity in the MEC server

## Implemented secured MEC model in VANET using blockchain-assisted deep learning: structural description

The system model of the VANET is clearly described in this section, and the description of the proposed model, including the secure MEC model in the VANET and its overall workflow flow is provided in this section. The details of the network parameters and the dataset details used for the implementation of the presented model are also given here.

### VANET: system model

VANETs establish a dynamic network for an intelligent transportation system. This system exchanges real-time information regarding road conditions, potential hazards, and vehicle positions, enhancing overall traffic safety and operational efficiency. Vehicles use Dedicated Short-Range Communication (DSRC) technology to send and receive data, enabling ad-hoc networking. This supports various applications, such as accident prevention and traffic control. The continuous data flow offers features like instant alerts for accidents or road obstructions, acting as a digital co-pilot to improve driving safety.

The system also creates engaging user experiences by integrating entertainment and information services. Moreover, it enhances the driving experience by promoting road safety. MEC reduce the latency, allowing faster data processing and quicker response times, which are crucial for real-time traffic control. MEC enables local data processing by reducing the strain on the broader network by analyzing data before forwarding it. As a result, only the most relevant data is transmitted, saving bandwidth and improving overall efficiency.

### Proposed secured MEC model in VANET: structural preview

The proposed secure MEC within VANET incorporates a range of robust cryptographic techniques and algorithms to enable efficient data processing and strengthen security. In this phase, the data is securely stored and authenticated for reliability within smart transportation systems. It starts by defining the essential VANET features required for authentication. In this work, the blockchain system is used for securing the information within the cloud structure. Here, the controlled authorized access among the participants is ensured by a permissioned blockchain. The strict access control for the vehicular networks is provided by this blockchain to facilitate transparency and trust. The proposed model uses the ADHyNet framework to process data from VANET nodes. Next, authenticity is validated in ADHyNet based on identified patterns. The RNU-SOA algorithm is designed to optimize various parameters of ADHyNet. This optimization fine-tunes these parameters to improve performance metrics, such as accuracy in assessing node trustworthiness. These metrics are essential for securing communications and preventing fraudulent behaviour within the network, ultimately enhancing vehicular safety and system integrity.

In the final stages, the HECC technique is used to perform the encryption process. The HECC is designed by fusing Homomorphic Encryption and ECC. In HECC, the authorized node data undergoes Homomorphic Encryption. This first level of encryption, referred to as half-enc, allows certain operations on the data without decryption, preserving data confidentiality while enabling limited computations. The partially encrypted data from Homomorphic Encryption is further protected using ECC. This additional layer of encryption strengthens security by ensuring that only trusted entities can access the encrypted data.

Initially, the data is secured with ECC and then decrypted to recover the half-encrypted information. Afterwards, the Homomorphic encryption is decrypted to retrieve the original node data. This process ensures that the data is securely accessed and utilized while maintaining confidentiality through the encryption methods.

Therefore, this cryptographic strategy effectively combines the benefits of two encryption techniques, creating a secure framework for managing sensitive information in vehicular networks. It promotes both confidentiality and data usability. A thorough evaluation is necessary to confirm the feasibility and effectiveness of the proposed approach in enhancing the overall security of vehicular networks.

### Initialization of network parameters

In this research work, essential information needed for the validation is collected from the dataset named Dataset-of-vehicular-communications-traces and from the link https://github.com/Djamila-Zamouche/Dataset-of-VANET-communication-traces/tree/main Access Date: 2025–10-11. This dataset uses the data, which is collected from the real traffic scenario using dual software such as Network Simulator v. 3.29 and Simulation of Urban Mobility (SUMO). Here, the open street map is used with SUMO by validating the traffic regions up to 25 km. Nearly 300 vehicles are considered under the VANET environment, with 15 malicious vehicles to identify the abnormal activities. Different information presented in the dataset includes start transmission, end transmission, period, packet counts, debit and distance.

By integrating this VANET dataset under various security attacks, the MEC model is evaluated against such vulnerabilities. In this work, various VANET attributes such as vehicle status, behaviour, indicator and trust level are mapped. In addition, the consideration of vehicular attributes helps to perform the simulation under the condition of the vehicular scenario. The effectiveness of the recommended blockchain-based MEC framework for the secure data sharing and authentication approach is determined using the VANET attributes.

VANET attributes like node names, node position, topology, average response time, and average packet delivery rate are utilized for authentication purposes. Node trustworthiness score was synthetically generated based on attributes such as Consistency in route announcements, Reputation history over time, Frequency of message broadcasting, and Message drop ratio. These attributes were mapped to analogous features from the VANET dataset, such as transaction anomalies, access logs, and behavioral consistency.

The dataset is divided into two parts for the proposed model: 750 samples are allocated for training data, which accounts for 75% of the total dataset of 1000 samples, and 250 samples are reserved for testing data, making up the remaining 25%. The variables used in the paper and their role are illustrated in Table 2.

**Table 2:** Variables and their roles.

Variables	Description
$R_{i}$	Input for LSTM
$R_{o}$	Output from LSTM
$R_{f}$	Forget gate in LSTM
$\zeta$	Sigmoid activation function
$Z$	Weight matrix
$d$	Bias
$k$	Hidden state in LSTM
$b$	Feature vector of LSTM
$i$	Time step
$G_{u}$	Update gate of GRU
$G_{r}$	Reset gate of GRU
$k_{r - 1}$	Hidden state of GRU
$b_{u}$	GRU Input vector
$\rho$	GRU Activation function
$Z_{i}$	Weight matrix of GRU
$HN_{bj}^{LSTM}$	Optimized hidden neuron count of LSTM
$HN_{cv}^{GRU}$	Optimized hidden neuron of GRU
$Rl_{pg}^{LSTM}$	Optimized learning rate of LSTM
$Rl_{kb}^{GRU}$	Optimized learning rate of GRU
$K_{obj}$	Objective function
$Ca$	Accuracy
$FOR$	False Omission Rate
$E_{vp}$	True positive
$J_{vn}$	False negative
$J_{vp}$	False positive
$E_{vn}$	True negative
$Fr$	Random variable of SOA
$M_{Rt}$	Maximum iteration
$y$	Uniform variable selection ranges from 1 to 2
$V$	Population Size
$F_{dl}$	Decrypt function of Homomorphic encryption
$R_{ql}$	Encryption function of Homomorphic encryption
$dl$	Public key of Homomorphic encryption
$\left( {z1,\,z2} \right)$	Plain text data
$H_{s}$	Randomly selected ECC private key
$j$	Elliptical curve
$L_{s}$	Public key of ECC
$FC$	Specificity
$TS$	Sensitivity
$Np$	Precision
$FNR$	False Negative Rate
$FDR$	False Discovery Rate
$CSI$	Critical Success Index

**Fig. 1:**
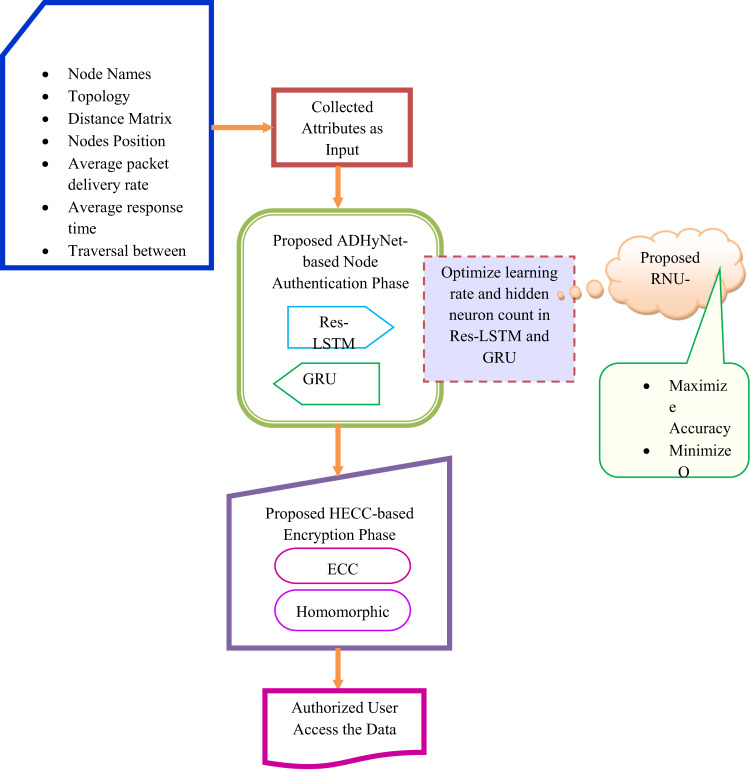
Diagrammatic representation of the proposed secured MEC model in VANET

The diagrammatic illustration of the developed secure MEC model in VANET is specified in Fig. 1.

## Trusted node authentication using dilated hybrid deep learning framework

The description of the ResLSTM model and GRU for designing the hybrid model to execute the authentication is elaborated here. The description of the Trusted Node Evaluation using Proposed ADHyNet and the parameter tuning in the ADHyNet via the RNU-SOA is also elaborated in this section.

### Residual long short-term memory

In this developed secured MEC model in the VANET environment, the Res-LSTM model is designed to analyze patterns in vehicular network data. Res-LSTM combines the architecture of LSTM with the residual connection.

LSTM^33^ consist of memory blocks with various gates like input $R_{i}$, output $R_{o}$, and forget gates $R_{f}$.

The input gate decides which information is added to the cell state using the formula in Eq. (1).

1 $$R_{i} = \zeta \left( {Z_{i} b_{i} + T_{i} k_{i - 1} + d_{i} } \right)$$

The output from the cell is controlled by the output gate using the formula in Eq. (2).

2 $$R_{o} = \zeta \left( {Z_{o} b_{o} + T_{o} k_{o - 1} + d_{o} } \right)$$

Forget gate: Remove the irrelevant data from the cell state using the formula in Eq. (3).

3 $$R_{f} = \zeta \left( {Z_{f} b_{f} + T_{f} k_{f - 1} + d_{f} } \right)$$

Here, the weighted matrix is indicated as $Z$ and $T$. The sigmoid activation function is denoted as $\zeta$. The bias term is represented as $d$, and the feature vector of the input state at time step $i$ is indicated as $b$. The term $k$ is the hidden state that controls the information flow within the network using the formula in Eq. (4).

4 $$k_{i} = \tan \left( {b_{i} } \right) \otimes R_{o}$$

LSTM networks face difficulties in effectively training the lower layers due to the vanishing gradient problem. To address this, residual connections are used to allow gradients to flow directly through the skip connections without increasing complexity. In Res-LSTM, the output of the LSTM cell is combined with the input of the existing layer through a skip connection, which facilitates better gradient flow during training. This structure consists of a stack of LSTM layers with residual connections, which helps retain essential information. Batch normalization is added at the end to improve network stability by reducing overfitting issues.

### Gated recurrent unit

The GRU network is chosen in this proposed model for managing the flow of information and retaining the essential features for long-term effectiveness over traditional RNNs.

GRU^32^ is composed of fewer gates than LSTM, which boosts the performance with less computational complexity and a faster training process. The update gate $G_{u}$ and reset gate $G_{r}$ are the two gating mechanisms performed in GRU.

*Update gate *$G_{u}$: This gate determines the quantity of the prior hidden state that should be retained and the amount of new data integrated into the network using the formula in Eq. (5).

5 $$G_{u} = \rho \left( {Z^{u} b_{u} + T^{u} k_{u - 1} } \right)$$

The term $b_{u}$ is the input vector, $\rho$ is the activation function and $Z$ and $T$ is the weight matrices of input and hidden state.

*Reset gate *$G_{r}$: It effectively identifies how much of the earlier inputs are mapped to the current input. Meanwhile, this gate decides how much of the previous information to disregard, modifiable how past data impacts the present hidden state. The reset gate is mathematically expressed in Eq. (6).

6 $$G_{r} = \rho \left( {Z^{r} b_{r} + T^{r} k_{r - 1} } \right)$$

When revising the hidden state $k_{r - 1}$, the reset gate gathers the past state that will be eliminated, whereas the update gate helps in merging past data with the current input to create the updated state. The hidden state $k_{r - 1}$ is generated by the GRU serves as an output for the classification task. The continuous modification of the hidden state through the gates allows the network to dynamically adapt to data patterns. As a result, GRUs offer significant advantages in processing sequential data, especially for non-linear tasks, due to their powerful gating system. This system improves memory retention and reduces the training challenges typically encountered with conventional RNNs.

### Trusted node evaluation using proposed ADHyNet

Trusted node evaluation using the proposed ADHyNet model integrates Res-LSTM with GRU, aiming to improve the security and reliability of nodes in a VANET. The initialized network attributes related to nodes, which are indicated as $N_{A}$ is fed to ADHyNet. The primary goal of ADHyNet is to evaluate and maintain the integrity and reliability of nodes within the VANET. This is crucial because VANETs are often vulnerable to threats from untrustworthy nodes, which can compromise data integrity and security. ADHyNet uses two main components: Res-LSTM and GRU.

*Res-LSTM* is an improved version of traditional LSTM networks. It incorporates residual connections that help with gradient flow during training, effectively addressing the vanishing gradient problem **t**hat typically arises in deeper networks. This enhancement allows the model to focus on long-term dependencies more efficiently. For trusted node evaluation, Res-LSTM identifies temporal patterns and trends in node activities over time, enabling a more accurate assessment of trustworthiness based on past interactions.

*GRU*, known for its simplicity and efficiency compared to LSTM, combines the input and forget gates into a single update gate, thereby minimize parameters counts. The performance of ADHyNet is further improved by dynamically tuning parameters based on observed data patterns, with the help of the RNU-SOA. The fitness function is designed to enhance authentication accuracy while minimizing FOR as shown in Eq. (7).

7 $$K_{obj} = \mathop {\arg \min }\limits_{{\left\{ {HN_{bj}^{LSTM} ,HN_{cv}^{GRU} ,Rl_{pg}^{LSTM} ,Rl_{kb}^{GRU} } \right\}}} \left( \frac{1}{Ca} \right) + FOR$$

Here, the terms $HN_{bj}^{LSTM}$ and $HN_{cv}^{GRU}$ are the optimized hidden neurons from both Res-LSTM and GRU that are selected in the range of [5–225]. The terms $Rl_{pg}^{LSTM}$ and $Rl_{kb}^{GRU}$ are the tuned learning rate from Res-LSTM and GRU that is selected in the interval of [0.01–0.99]. The model is configured with the following hyperparameters: initially, the suggested model is trained for 5 epochs, with each epoch consisting of 100 steps. The proposed architecture features 5 hidden neurons. A batch size of 4 is used for training, which allows the model to process small batches of data at a time. The Rectified Linear Unit (ReLU) activation function is employed to introduce non-linearity into the model. This configuration provides a foundation for training an effective model.

The accuracy *Ca* and FOR are evaluated using the formula in Eqs. (8) and (9).

8 $$Ca = \frac{{E_{vp} + E_{vn} }}{{E_{vp} + E_{vp} + J_{vp} + J_{vn} }}$$

9 $$FOR = \frac{{J_{vn} }}{{J_{vn} + E_{vn} }}$$

In this phase, the true positive and negative rates are denoted as $E_{vp}$ and $E_{vn}$. The false positive and negative values are represented as $J_{vp}$ and $J_{vn}$, respectively. Additionally, dilated mechanisms are utilized to broaden the receptive field of the neural network, enabling the ADHyNet to gather information from a more extensive range of contexts without significantly raising computational demands. This feature is particularly advantageous in situations where insights from distant time steps (or interactions) are required to perform trust evaluations.

*“Hybrid" and “dilated” aspects of the network architecture and their relevance to VANETs*: The integration of computational techniques and multiple security techniques is represented as a “hybrid” aspect in the proposed research work. In this work, the deep learning models such as GRU, Res-LSTM and ADHyNet, along with the blockchain technology, are hybridized for providing security to the VANET. Here, the deep learning models can be used for the effective detection of malicious activities in the VANET. In addition, decentralized trust and immutable data logging are offered by the integration of blockchain technology.

In the deep learning model, the “dilated” mechanism is used, which can expand the receptive field of the network. The enlargement in the receptive field does not improve the computational complexity, and also effectively captures the long-range dependencies of the data. The timely detection of vehicular communication is more crucial, so capturing the long-range dependencies is more relevant for the VANET. The threats and the anomalies under the changing environmental conditions are effectively captured through a better understanding of the spatial and temporal patterns within the network. In the fast-moving vehicular environment, analyzing the network behaviour is important, which is more effectively performed through a dilated mechanism. Moreover, the security standard in the VANET is improved via the hybrid architecture, as it can easily adapt to the network states and attack types in the VANET.

**Fig. 2:**
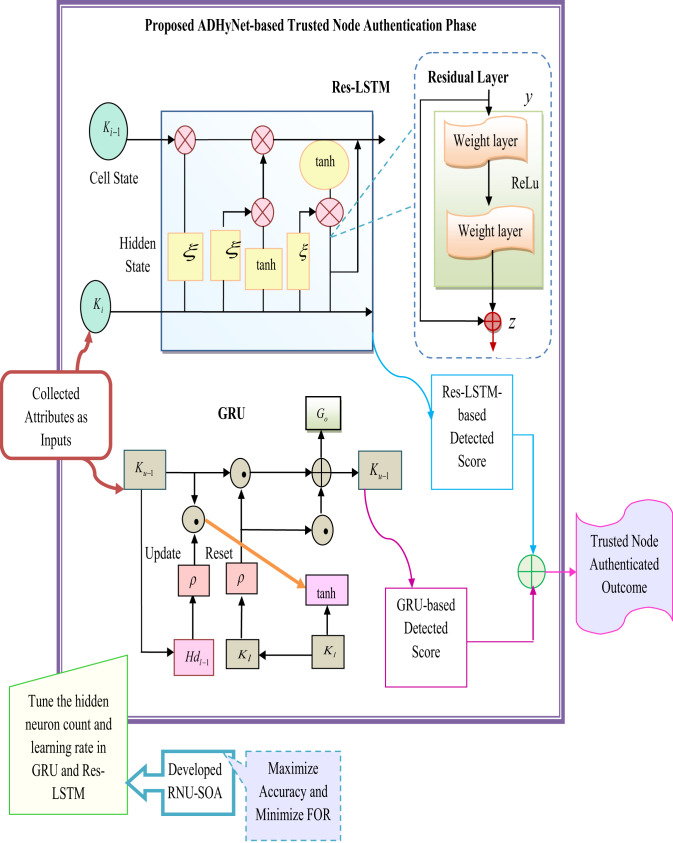
Visual representation of proposed ADHyNet-based trusted node authentication process

*Trusted node authentication procedure*: ADHyNet collects data from multiple nodes, each with various attributes. Using the combined framework of Res-LSTM and GRU, the network analyzes this data to detect patterns that indicate whether nodes can be trusted. Here, the node-detected outcomes are attained from Res-LSTM and GRU. Next, the two sets of detected outcomes are averaged and offered as the trusted node authentication outcomes. The result is a trust score or assessment for each node, which reflects its reliability based on past behaviour. This score helps guide future decisions within the VANET. By accurately assessing node trustworthiness, ADHyNet helps mitigate the risks posed by malicious nodes. The model’s adaptive and hybrid nature ensures its flexibility in response to changing network conditions. Trusted node evaluation using the proposed ADHyNet is diagrammatically illustrated in Fig. 2.

### Hyperparameter adjustment using RNU-SOA

Skill Optimization Algorithm (SOA)^2^ is an innovative human-inspired metaheuristic approach aimed at addressing optimization issues. During this phase, SOA participants (which represent possible solutions) strive to gain skills with the support of expert members who possess superior objective function values. These participants are encouraged to thoroughly explore the search space, influenced by various members instead of concentrating on the best solution. Initially, the input parameters relevant to the optimization function are defined and randomly initialized as the position of SOA. After initialization, the objective function for each solution is computed based on its fitness values. Yet, if the exploration phase of SOA achieves less convergence, then SOA takes more time to optimize the solution. The effectiveness of SOA is reduced due to the complex search space. To overcome these challenges, an SOA is modified into RNU-SOA by updating the random variable $Fr$ of SOA.

Novelty of RNU-SOA: In the proposed RNU-SOA, the random number $Fr$ is updated throughout the optimization process. By vigorously adjusting the $Fr$ RNU-SOA increases its ability to explore new areas of the solution space. This adaptability helps to prevent the method from getting stuck in local optima, particularly in complex optimization. By avoiding local optima and maintaining diversity, RNU-SOA achieved better-quality solutions. Random variable *Fr* in SOA is updated using Eq. (10).

10 $$Fr = - y \times \frac{0.02}{{M_{Rt} }}$$

Here, the term $M_{Rt}$ is the maximum iteration and *y* is the variable, which is randomly selected from^1,2^. This modification boosts the performance of RNU-SOA in solving complex optimization problems. The Pseudocode of RNU-SOA is given in Algorithm 1.

**Fig. 3:**
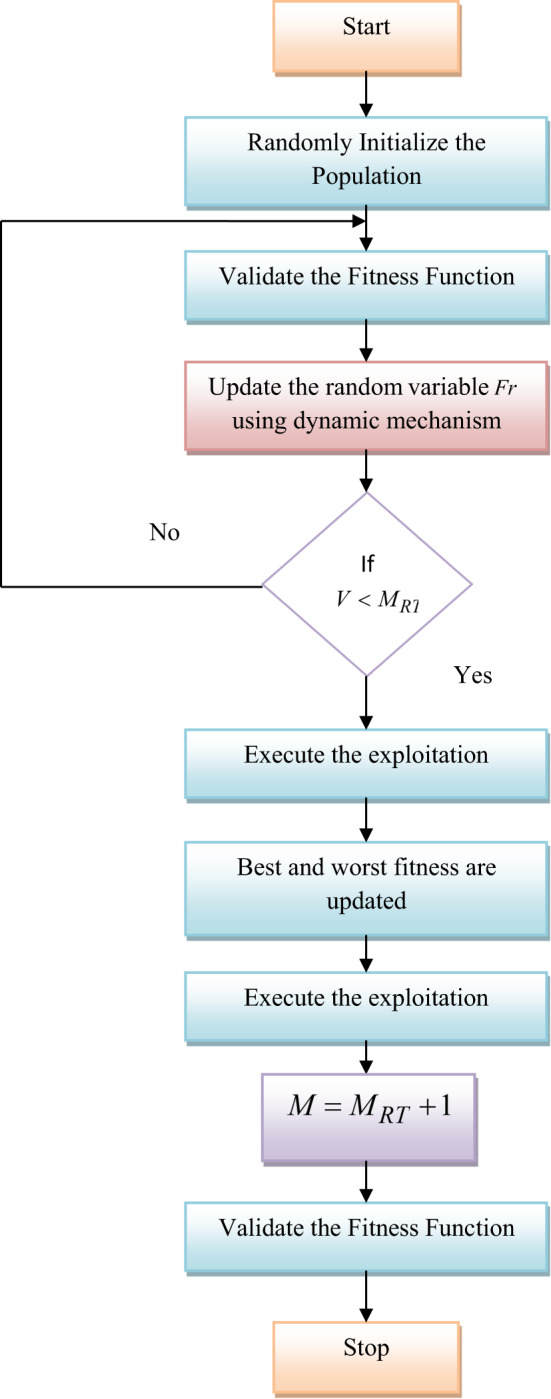
Flowchart of proposed RNU-SOA model

The Flowchart of RNU-SOA is provided in Fig. 3.

## Blockchain-based secure data storage using encryption algorithms in MEC

The details of the encryption approaches in the proposed research are elaborated in this section. The working of the hybrid encryption developed with the combination of the conventional encryption approach as well as the storage of the encrypted data in the blockchain network, is also given in this section.

### Homomorphic encryption

Homomorphic encryption^34^ is a form of encryption that permits calculations to be executed directly on encrypted data, allowing the processing of ciphertexts without decrypting the data.

*Partial Homomorphism Encryption (PHE)*: It enables either addition or multiplication operations, but not both simultaneously. Additive Homomorphic Encryption (AHE) specifically allows for addition operations to be conducted on ciphertexts using Eq. (11).

11 $$F_{dl} \left( {R_{ql} \left( {z1} \right) \cdot R_{ql} \left( {z2} \right)} \right) = z1 + z2$$

Here, the term $F_{dl}$ is the decrypt function and $R_{ql}$ is the encrypt function. The public key is denoted as $dl$ and the plain texts are indicated as $\left( {z1,\,z2} \right)$.

*Fully Homomorphic Encryption (FHE)*: It allows for performing addition and multiplication operations on encrypted data without decrypt it. In terms of data security and privacy, the MEC model has become increasingly significant in edge computing environments. Edge devices can process sensitive data, such as personal information from mobile devices or Internet of Things sensors, without exposing it. This is made possible by homomorphic encryption. Homomorphic encryption greatly enhances the security architecture of MEC settings. It reduces the likelihood of data breaches and unauthorized access.

### Elliptic curve cryptography

Elliptic Curve Cryptography (ECC) is a public key cryptographic approach that uses the algebraic structure of elliptic curves defined over finite fields. It allows for the use of smaller key sizes compared to RSA, while maintaining equivalent security levels. This reduction in key size leads to decreased computational demands and lower memory requirements, making ECC particularly advantageous in resource-constrained environments. Each bit of the ECC key size provides a high level of security. The mathematical processes involved in ECC are less computationally intensive than those in RSA, which results in faster performance. This makes ECC ideal for devices with limited processing power and battery life.

*Mechanisms*: In ECC, the private key is randomly chosen and indicated as $H_{s}$. This key remains secret and is essential for tasks related to digital decryption. The public key is generated from the private key, calculated by multiplying a specific point calculated from the elliptical curve, and it is denoted as $L_{s} = H_{s} * j$. The public key $L_{s}$ is shared with others, enabling them to encrypt messages. The key generation process involves creating a pair consisting of a private key and a public key. Key sharing refers to the secure distribution of the public key, ensuring that the private key remains secret. Each participant generates their own pair of keys (private and public) and then exchanges public keys through a potentially insecure communication channel. Once the keys are exchanged through the key-sharing mechanism, participants can use their private keys to sign messages. The shared secrets obtained during the key exchange are then used to create symmetric keys for message encryption. These symmetric keys are applied through standard encryption techniques to protect the communication channel.

### Data encryption using HECC

HECC integrates the concepts of homomorphic encryption and ECC to perform computations on encrypted data. The step-by-step encryption process used in this approach is outlined below.

- In the first phase, the plaintext that needs protection undergoes half-encryption using a homomorphic technique. This type of encryption allows mathematical operations to be executed on the data without revealing the plaintext.
- The half-encrypted data from the previous step is then further secured using ECC. This step enhances security by encrypting the already encrypted data, ensuring that even if an unauthorized individual gains access to the half-encrypted data, they will be unable to decipher it without the correct ECC decryption keys.
- Finally, to access the original plaintext, the fully encrypted data from Step 2 must be decrypted using the ECC method. This operation yields the half-encrypted data. Once the half-encrypted data is obtained, it is processed through the homomorphic decryption function, resulting in the retrieval of the original plaintext.

**Fig. 4:**
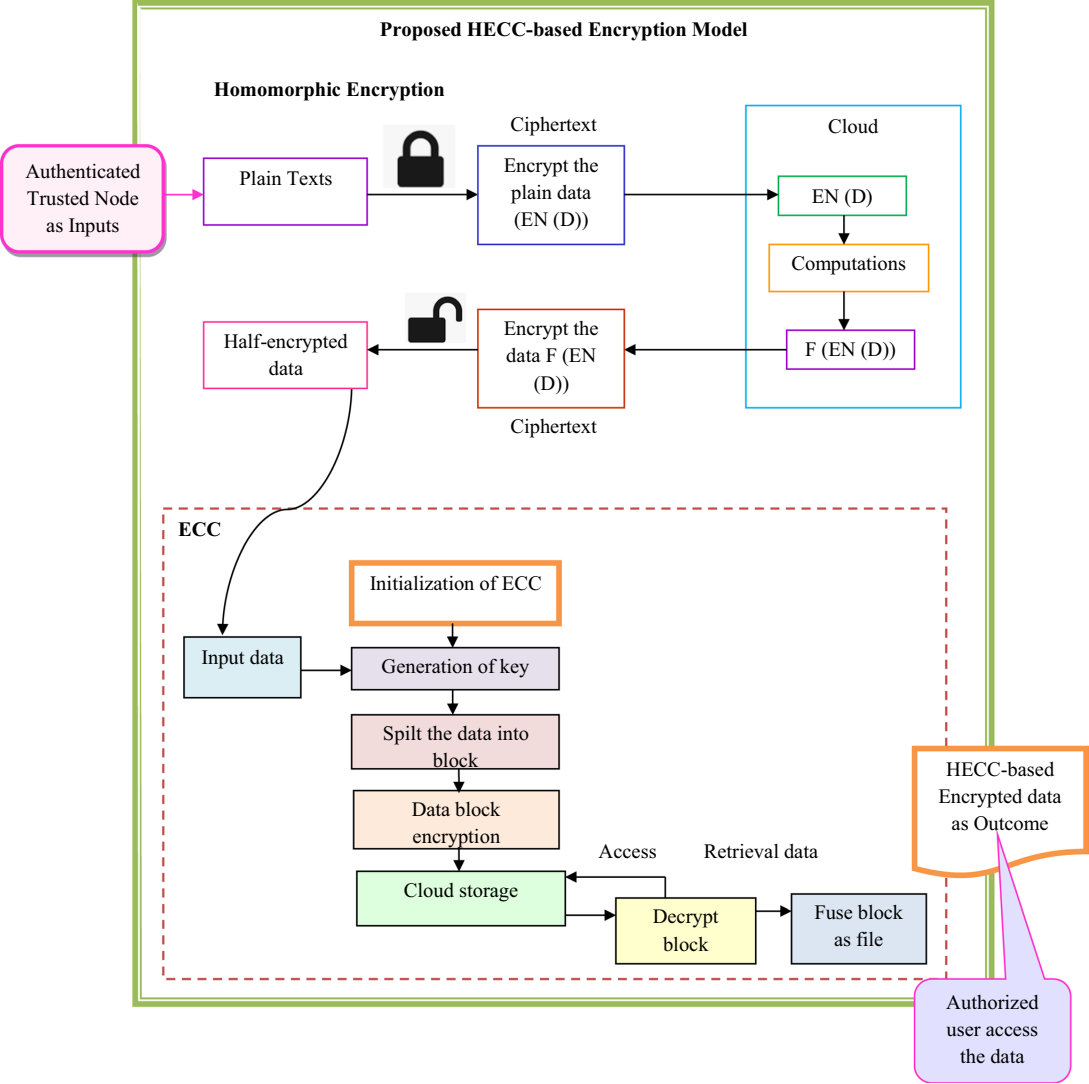
Diagrammatic representation of data encryption using HECC

This entire procedure ensures that data remains secure throughout the computation process. Data encryption using HECC is visually represented in Fig. 4.

*Proof-of-security-of-security in Developed HECC*: This section explains the proof-of-security in the developed HECC-based data encryption model. Here, the developed HECC-based data encryption model hybridized the strengths of ECC and homomorphic encryption to improve the security of the network. The developed cryptographic technique HECC is better at managing the data from various attacks by fulfilling various security measures. Moreover, the developed scheme uses the blockchain to preserve the sensitive information about the nodes in the VANET. In this phase, the census technique is employed to verify the transaction process in the blockchain. Here, the ECC is employed to maintain robustness with better key generation at the initial encryption phase, and then homomorphic encryption is carried out in the ciphertext. The ECC is good at maintaining security against various attacks and also effectively tackles the elliptic curve discrete logarithm problems. Next, the homomorphic encryption model is efficient in tackling the lattice issues and also effectively reduces the errors. Moreover, the integer factorization issues in the encryption phase are tackled. Moreover, the confidentiality of the developed HECC is improved as it didn’t showcase the sensitive information presented in the plaintext. In addition, the developed model displayed that the validations carried out on the ciphertext are widely suitable for similar operations carried out in the plaintext. The developed HECC is good at accomplishing better outcomes by reducing the performance overheads. This technique is suitable for carrying out limited functionality such as multiplication and addition operations.

### Blockchain-based data storage for security

Blockchain operates on a decentralized network of nodes, helping to distribute data across various nodes. Once information is registered on the blockchain, it cannot be modified. All users within the blockchain network can access the same information. Data stored on the blockchain is protected by advanced cryptographic methods. Each data entry is encrypted and controlled using private and public keys, providing an added layer of security against unauthorized access. Since data is duplicated across multiple nodes, blockchain systems recover more effectively from failures or data loss compared to traditional systems. If a single node fails, other nodes still retain the entire historical record of the data.

**Fig. 5:**
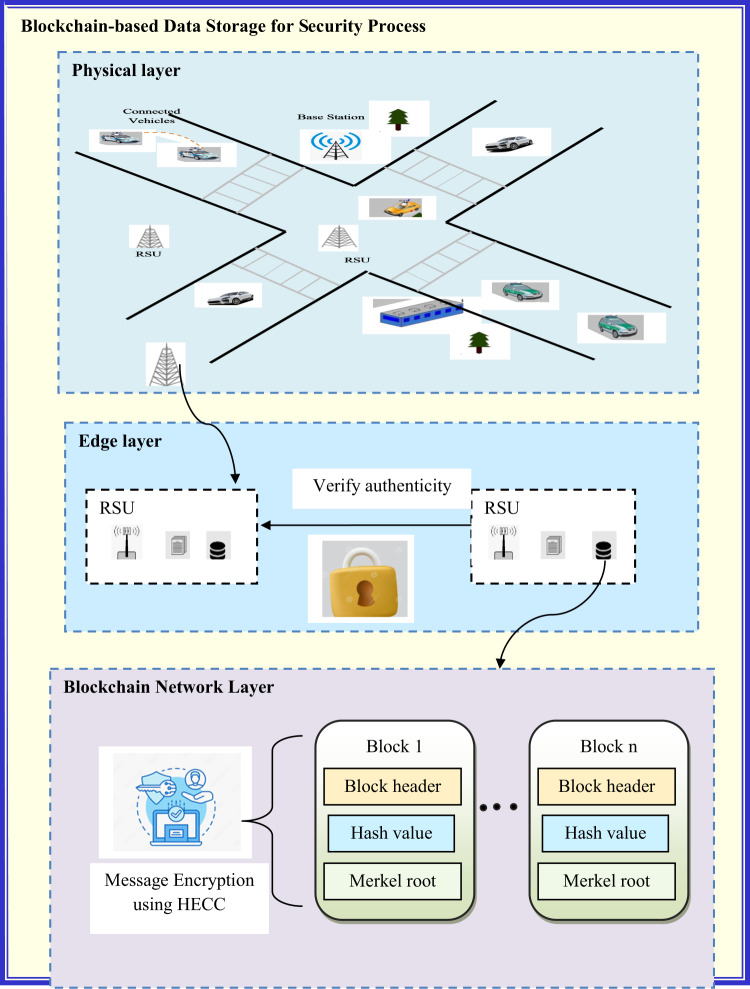
Diagrammatic representation of blockchain-based data storage for security process

Blockchain-based data storage creates a secure environment for managing sensitive information by using cryptographic techniques and consensus mechanisms to maintain data integrity and protect user privacy. This ensures that data is securely stored and managed within the VANET system, improving the efficiency of the secured MEC model in the VANET system. Blockchain-based data storage for the security process is diagrammatically shown in Fig. 5.

The proposed framework aims to establish a blockchain-powered deep learning architecture to guarantee the safety and privacy of VANETs. The framework is structured into three primary layers: perception, edge computing, and services, each contributing to the overall security and functionality of the network.

- *Perception Layer*: This layer primarily focuses on protecting the privacy of VANET data by managing blockchain activities. The blockchain ensures transparent, immutable logging of vehicle data, which enhances trust and prevents unauthorized modifications.
- *Edge Computing and Cloud Service Layer*: This layer processes and computes data using edge computing techniques along with cloud services. It also interacts with the blockchain to ensure that data processing and storage are secure and enable real-time decision-making.
- *Service Layer*: This layer handles data encryption and privacy protection by leveraging the blockchain and maintaining information within a public cloud. Blockchain ensures that only authorized parties can access and modify sensitive data. Additionally, HECC is used to secure data from unauthorized access during storage and transmission.

The final goal of the model is to assess and ensure the dependability of vehicle nodes on the blockchain, particularly through the use of ADHyNet for node authentication. Res-LSTM, integrated with GRU, forms the core of ADHyNet, while RNU-SOA optimizes the hyperparameters for better performance.

The role of blockchain in the developed three-layer architecture has been effectively described in the above points. It is not merely a decentralized process but an integral component for data security, privacy protection, and ensuring dependability through immutable logging of vehicle node information. Blockchain facilitates transparency and security within the system, particularly for node authentication, task offloading, and service provisioning.

The blockchain consensus details are listed in Table 3.

**Table 3:** Blockchain consensus details.

Parameters	Values
No of Sub Carriers	^2,4,6,8,10,12^
Number of Nodes	50, 100, 150
Total Bandwidth	5bps
System Delay requirement	1
Circuit power	30 J
Antenna Power	27 J
Power Amplifier Coefficient	0.38
Maximum No of Users	50
Initial Energy	0.5 J
d1%Distances (d1,d2)	500m; 200m
Power allocation coefficients (a1, a2)	0.75; 0.25
Path loss exponent	4;%
Bandwidth	10^6^ bps
Choice of consensus protocol	PoS

## Results and discussion

This section provides the information regarding the simulation environment and metrics used for the validation process. Authentication performance analysis, cryptography performance analysis, total computational time analysis, statistical analysis numerical evaluation of authentication performance is elaborated in Section "Results and discussion".

### Experimental setup

The proposed secure MEC framework in the VANET system using blockchain was implemented in Python. VANET attributes were initialized under different conditions to validate the robustness and efficiency of the secure MEC model against various attacks. The reliability of the developed framework was compared with existing methods to improve network throughput during simulation. The proposed Blockchain-based Secure MEC Model is implemented using Python 3.11 on a Windows 11 Pro system with a 64-bit operating system, running on an Intel Core i3-1005G1 CPU @ 1.20 GHz with 16 GB of RAM. The system utilizes integrated Intel UHD Graphics. Given the specifications, the model would likely leverage the capabilities of blockchain technology, such as decentralization, immutability, and transparency, to ensure secure and efficient data processing in a Mobile Edge Computing (MEC) environment.

Existing algorithms, such as the Crystal Structure Algorithm (CSA)^26^, Fireworks Algorithm (FA)^27^, Northern Goshawk Optimization (NGO)^28^, and Skill Optimization Algorithm (SOA)^25^, were used for comparison. Techniques like AD-GRU^1^, Res-LSTM^2^, GRU^32^, and ADHyNet were employed to validate the efficiency of the proposed authentication model. Additionally, cryptographic models like Data Encryption Standard (DES)^30^, Advanced Encryption Standard (AES)^29^, Homomorphic Encryption^31^, and ECC were used for comparing encryption and decryption performance.

Simulation parameters used in the developed MEC model for VANET are offered in Table 4.

**Table 4:** Simulation parameters of the developed MEC model for VANET.

Parameters	Symbol	Recommended value
Network Size	N	200 Vehicle Nodes
MEC Nodes	M	10
Mobility Speed	V	10–30 m/s
Communication Range	R	300 m

### Performance metrics

The formula used for the classification task to assess the effectiveness is provided in the points below.

Critical Success Index (CSI) of the developed approach is assessed using the formula in Eq. (12).

12 $$CSI = \frac{Evp}{{Evp + Jvp + Jvn}}$$

The False Discovery Rate (FDR) of the developed approach is validated using the formula in Eq. (13).

13 $$FDR = \frac{Jvp}{{Evp + Jvp}}$$

False Negative Rate (FNR) of the developed approach is assessed using the formula in Eq. (14).

14 $$FNR = \frac{Jvn}{{Evp + Jvn}}$$

False Omission Rate (FOR) is calculated using the formula in Eq. (15).

15 $$FOR = \frac{Jvn}{{Evn + Jvn}}$$

Precision $Np$ is measured through Eq. (16).

16 $$Np = \frac{Evp}{{Evp + Jvp}}$$

Sensitivity $TS$ is measured using Eq. (17).

17 $$TS = \frac{Evp}{{Evp + Jvn}}$$

Specificity $FC$ is measured using Eq. (18).

18 $$FC = \frac{Evn}{{Evn + Jvp}}$$

### Authentication performance analysis

**Fig. 6:**
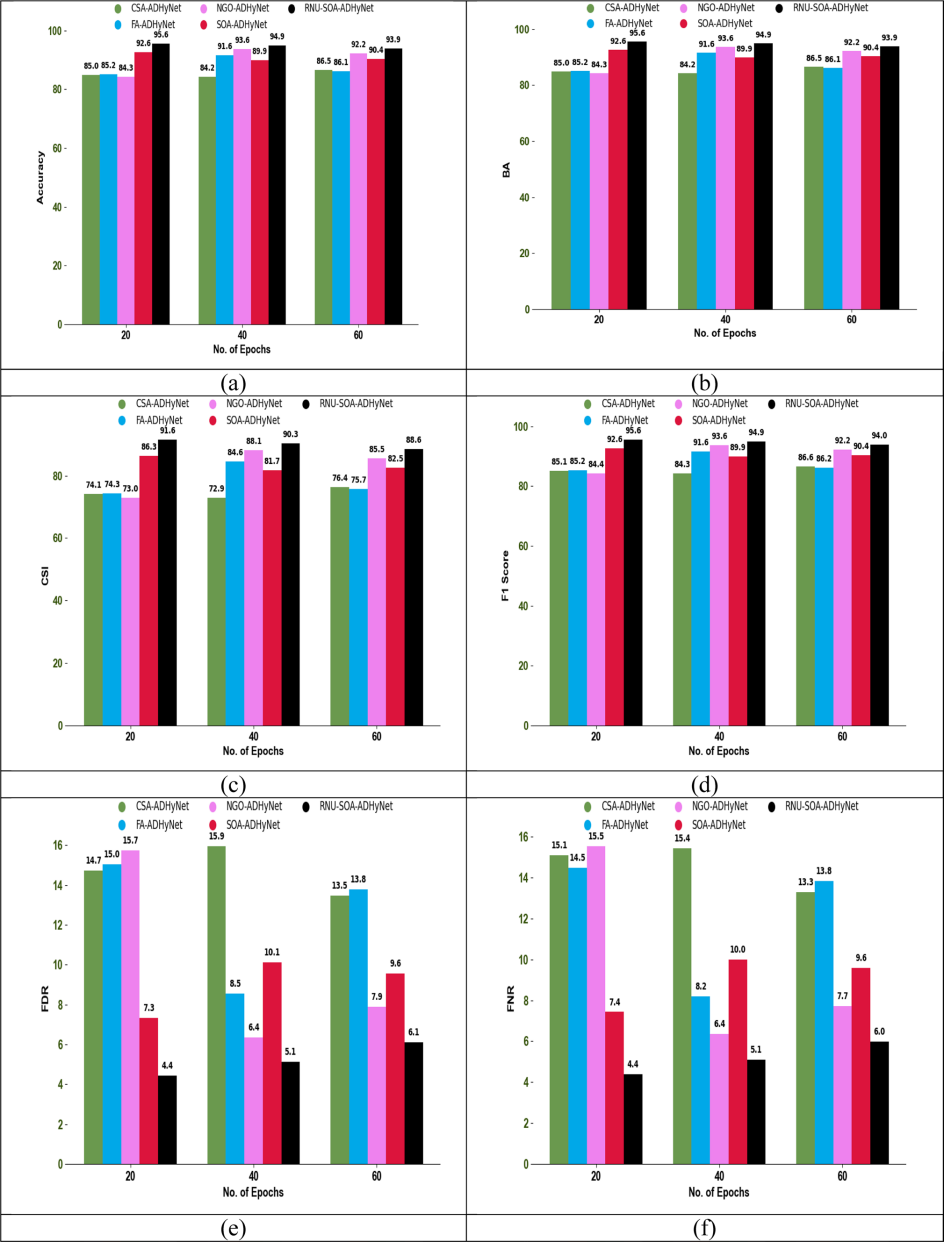

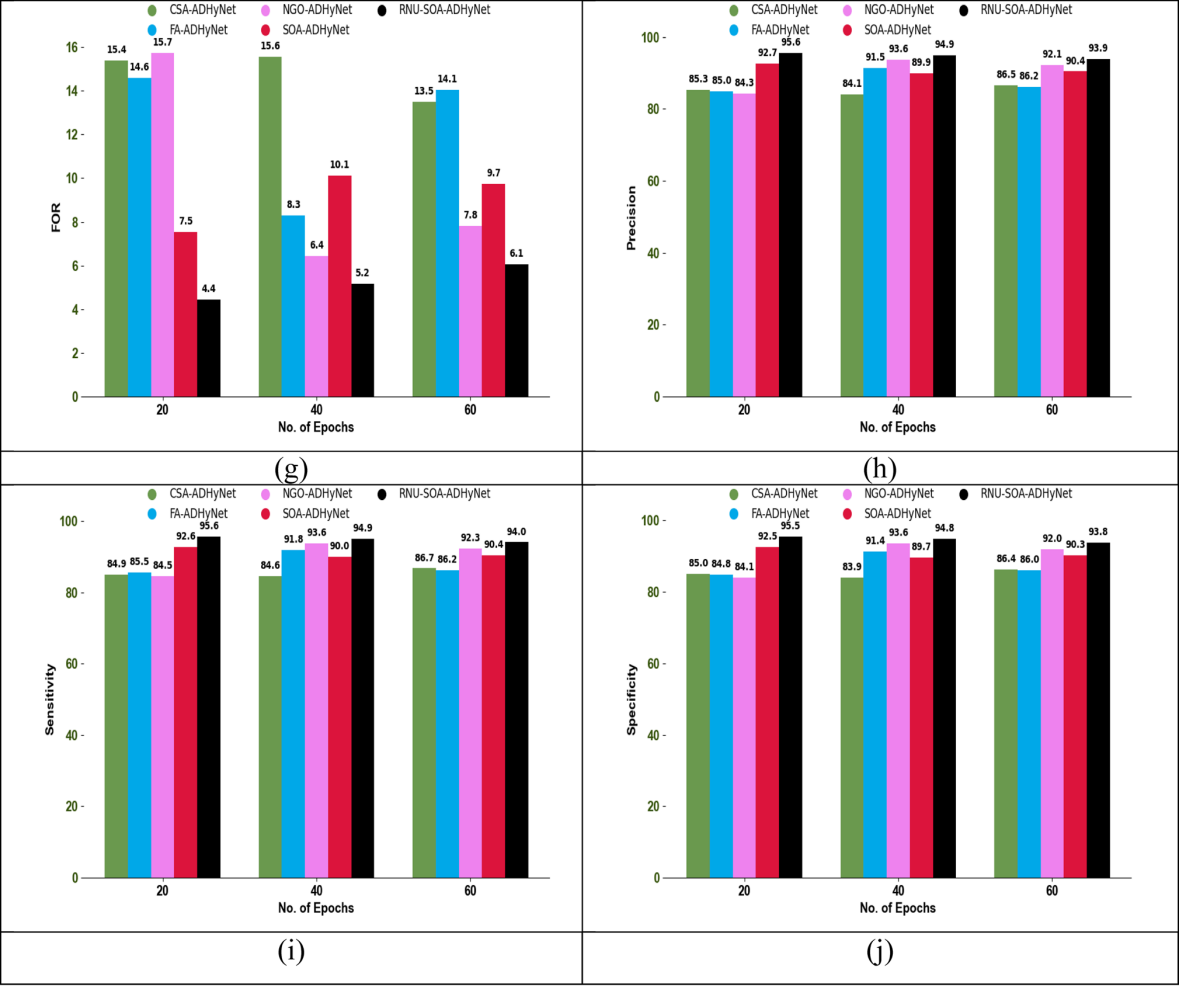
Optimization for authentication performance analysis among traditional algorithms regarding (**a**) Accuracy, (**b**) BA, (**c**) CSI, (**d**) F1-score, (**e**) FDR, (**f**) FNR, (**g**) FOR, (**h**) Precision, (**i**) Sensitivity, and (**j**) Specificity

**Fig. 7:**
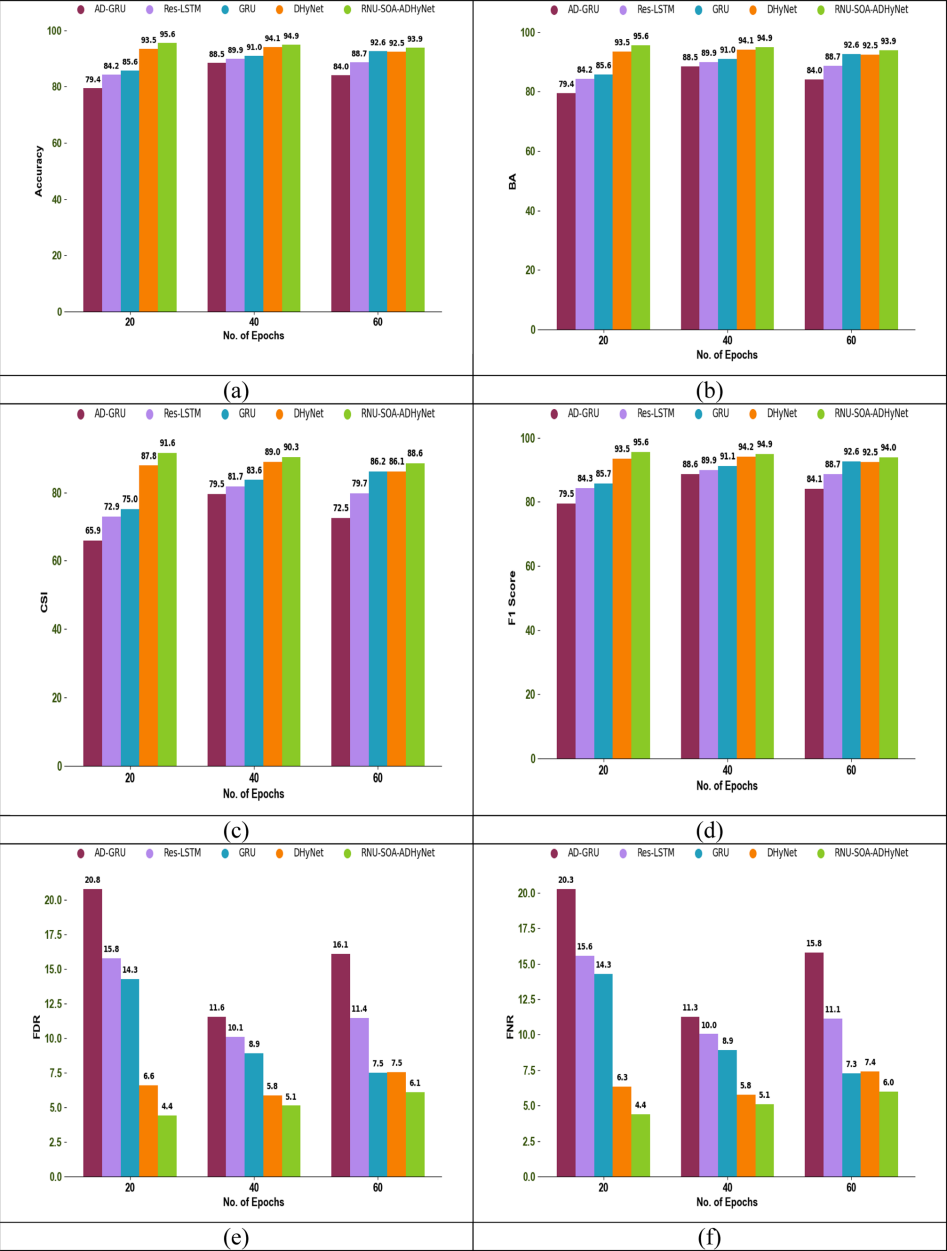

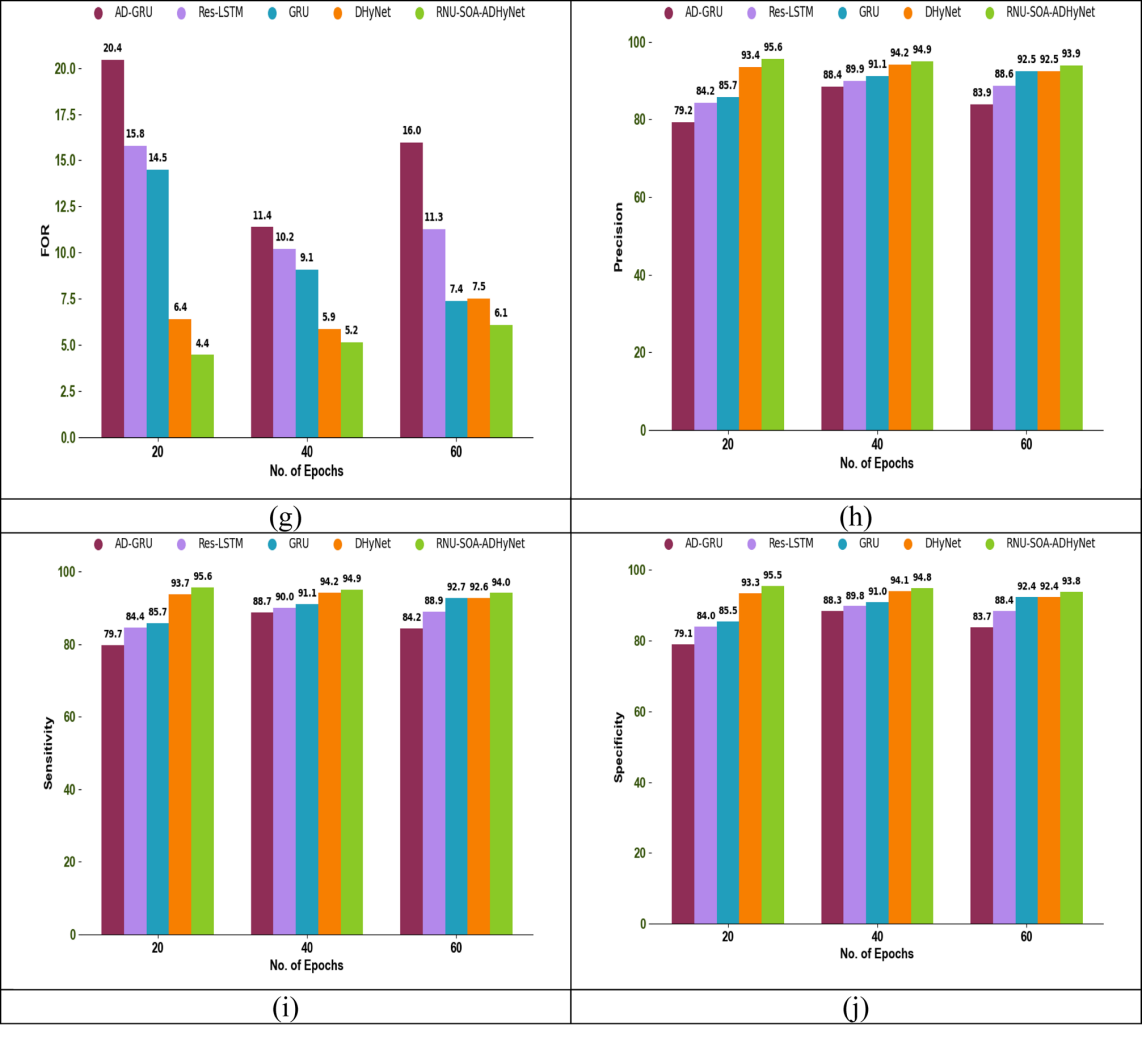
Optimization for authentication performance analysis among traditional techniques regarding (**a**) Accuracy, (**b**) BA, (**c**) CSI, (**d**) F1-score, (**e**) FDR, (**f**) FNR, (**g**) FOR, (**h**) Precision, (**i**) Sensitivity, and (**j**) Specificity

The authentication performance analysis using different performance metrics among existing algorithms and techniques is shown in Figs. 6 and 7, respectively. In this secure node authentication system, the performance of the proposed RNU-SOA-ADHyNet model is compared with existing models by varying the epoch count. By observing various performance metrics across different epoch counts, an optimal point where the proposed RNU-SOA-ADHyNet model achieves the best performance without overfitting.

As seen in Fig. 6a, the accuracy of implemented RNU-SOA-ADHyNet is 95.6%, which is 12.4% higher than the existing CSA-ADHyNet and 12.2% higher than the FA-ADHyNet model when the epoch count is set to 20. In the technical comparisons shown in Fig. 7a, the accuracy of our RNU-SOA-ADHyNet model is 95.6%, which is 20.4%, 13.5%, 11.6%, and 2.2% better than AD-GRU, Res-LSTM, GRU, and DHyNet, respectively, for an epoch count of 20.

The acceptable level of performance with the minimum epoch count will be analyzed using this performance analysis. The efficiency of the RNU-SOA-ADHyNet framework is enhanced by reducing the training time and computational resources. Therefore, the proposed RNU-SOA-ADHyNet model maintains its success rate over a range of epochs and is more reliable in dynamic environments, such as VANETs, where conditions frequently change.

### Cryptography performance analysis

**Fig. 8:**
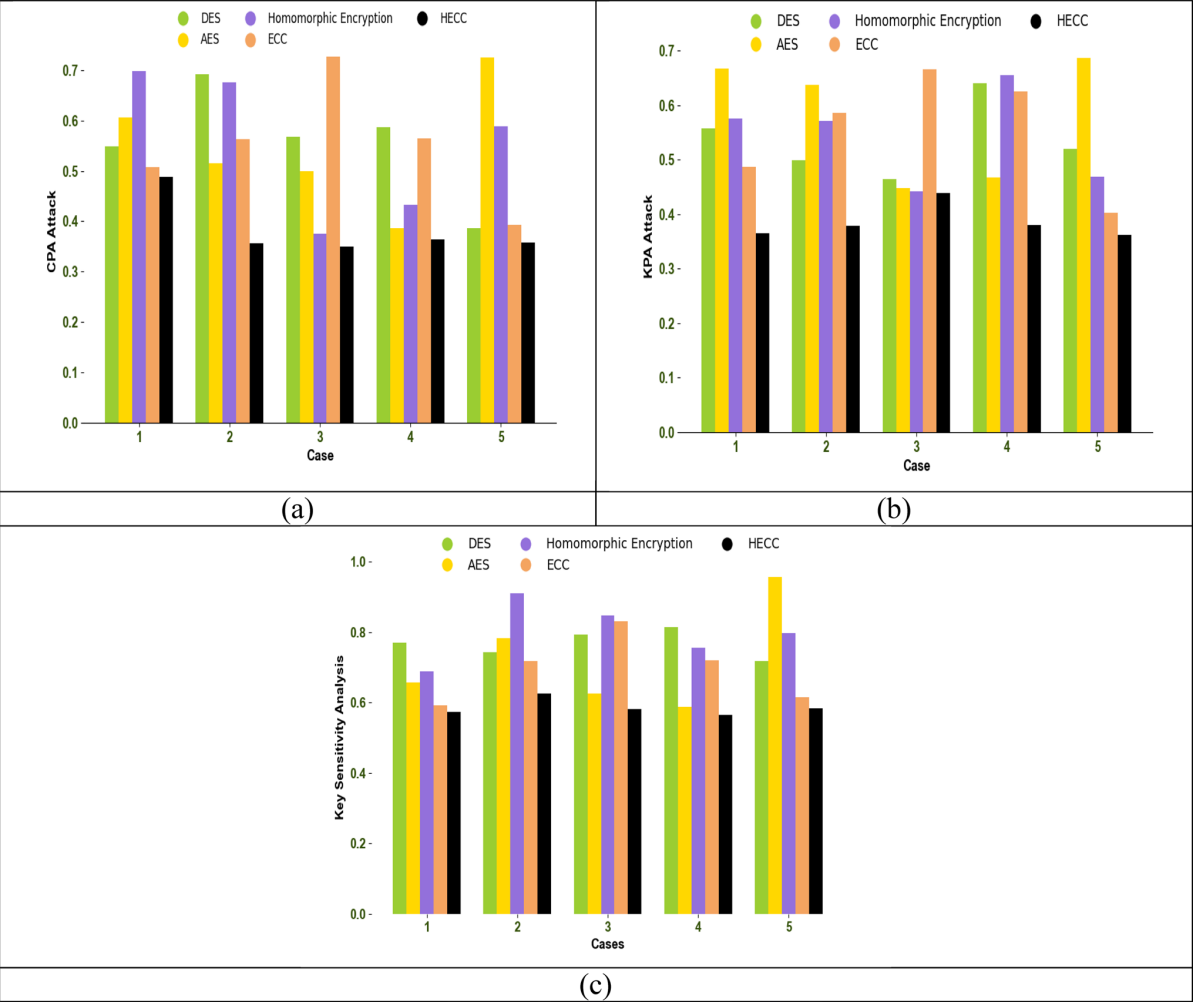
Cryptography performance analysis among traditional techniques regarding (**a**) CPA attack, (**b**) KPA attack, and (**c**) Key sensitivity analysis

Cryptography performance is analyzed in terms of Chosen-Plaintext Attack (CPA) and Known-Plaintext Attack (KPA), and the results are shown in Fig. 8. Evaluating performance based on a CPA attack helps assess the flexibility of an encryption scheme and whether any modifications in the input data can be detected. By varying the number of cases, the developed HECC approach outperforms existing techniques, demonstrating its robustness and flexibility in encryption schemes. The CPA attack rate of the proposed HECC model is reduced by 5.7%, 18.3%, 30%, and 2% compared to DES, AES, homomorphic encryption, and ECC for case 1.

By validating the encryption scheme based on a CPA attack, the proposed HECC scheme shows a strong ability to handle modifications in input data. The highly efficient HECC encryption scheme demonstrates minimal variations in the output even when the input data changes. Therefore, a lower CPA attack rate indicates that the HECC model is more flexible to chosen plaintext attacks compared to previously establish cryptographic models.

### Total computational time analysis

**Fig. 9:**
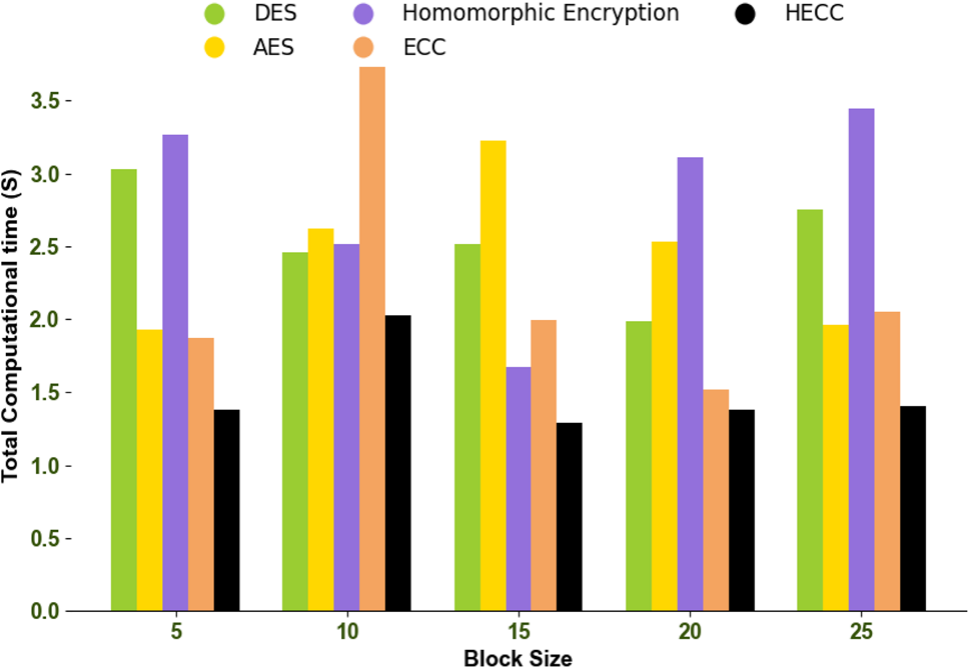
Total computation time analysis among traditional techniques

The computation time analysis of the developed framework is provided in Fig. 9. The maximum cumulative time taken by the developed system to perform its operation is referred to as computation time. The computational complexity of the implemented HECC model is 53.3%, 26.3%, 57.5%, and 22.2% lower than other traditional models like DES, AES, homomorphic encryption, and ECC. With reduced processing time, the HECC model can handle more complex operations. Thus, the low computation time helps the proposed HECC model to validate the data quickly and provide an immediate response in VANET.

### Statistical analysis

Statistical analysis using performance metrics of the hybrid MEC model is provided in Table 5. The reliability of the data is tested based on statistical analysis. The mean value of the designed RNU-SOA-ADHyNet model is minimized by 26.1% compared to CSA-ADHyNet, 30.7% compared to FA-ADHyNet, 21.2% compared to NGO-ADHyNet, and 26.2% compared to SOA-ADHyNet. The reductions in the mean values indicate that RNU-SOA-ADHyNet outperforms the compared models.

**Table 5:** Statistical Analysis of the proposed secure MEC model in VANET using traditional Algorithms.

Metrics	CSA-ADHyNet ^26^	FA-ADHyNet ^27^	NGO-ADHyNet ^28^	SOA-ADHyNet ^25^	RNU-SOA-ADHyNet
Standard Deviation	0.310832	0.622579	0.119649	0.270022	0.062477
Best	1.27641	1.343747	1.300357	1.334113	1.051198
Mean	1.452837	1.548636	1.361037	1.453692	1.072407
Median	1.296959	1.358383	1.336888	1.378046	1.051198
Worst	2.847881	4.062641	2.142458	3.126485	1.259685

Lower average values suggest that the RNU-SOA-ADHyNet model requires fewer resources while achieving better performance levels. By analyzing how resources are utilized in the MEC model statistically, efficient resource management is ensured in the proposed model. This optimization is critical for ensuring high performance without overloading the network. Thus, in the VANET environment, the proposed RNU-SOA-ADHyNet model achieves low latency and improved throughput, resulting in quicker response times and more consistent communication.

### Numerical evaluation of authentication performance

The validation of authentication performance employed a K-fold cross-validation that split the dataset into 5 different K-fold values. The numerical values of performance metrics among traditional optimization methods and techniques are illustrated in Tables 6 and 7, respectively. The detailed information in these tables validates how different algorithms perform under the same conditions, emphasizing the robustness and superiority of the proposed model.

**Table 6:** Numerical Analysis of the proposed secure MEC model in VANET using traditional Algorithms.

K-fold value	CSA-ADHyNet ^26^	FA-ADHyNet ^27^	NGO-ADHyNet ^28^	SOA-ADHyNet ^25^	RNU-SOA-ADHyNet
Accuracy					
1	74.85333	80.4	85.62667	86.4	90.61333
2	84.64	81.06667	88.24	90.66667	90.74667
3	81.49333	80.26667	81.09333	93.06667	95.09333
4	85.68	82.64	87.89333	89.17333	90.88
5	86.18667	86.74667	82.34667	86.34667	93.57333
Specificity					
1	74.29333	79.87186	85.40657	86.01286	90.55076
2	84.50324	80.75269	88.0043	90.43011	90.5762
3	81.1828	79.94624	80.86253	92.98813	94.9408
4	85.23274	82.25201	87.63441	88.96663	90.73276
5	85.83691	86.3807	81.90578	86.07527	93.61817
FPR					
1	25.70667	20.12814	14.59343	13.98714	9.449244
2	15.49676	19.24731	11.9957	9.569892	9.423802
3	18.8172	20.05376	19.13747	7.011866	5.059203
4	14.76726	17.74799	12.36559	11.03337	9.267241
5	14.16309	13.6193	18.09422	13.92473	6.381828
NPV					
1	75.13484	80.6904	85.54477	86.56958	90.45307
2	84.41208	81.01402	88.24164	90.72276	90.72276
3	81.44552	80.20496	80.90615	92.98813	95.14563
4	85.92233	82.74002	87.91802	89.15858	90.83064
5	86.29989	86.8932	82.52427	86.35383	93.3657
MCC					
1	0.497098	0.608027	0.712503	0.728	0.812241
2	0.692758	0.621303	0.76478	0.813321	0.814914
3	0.629837	0.605301	0.621822	0.861316	0.901859
4	0.713613	0.65279	0.757849	0.783446	0.81758
5	0.723725	0.73493	0.646937	0.726913	0.87145

**Table 7:** Numerical Analysis of the proposed secure MEC model in VANET using traditional techniques.

K-fold value	AD-GRU ^1^	Res-LSTM ^2^	GRU ^32^	DHyNet	RNU-SOA-ADHyNet
Accuracy					
1	79.52	81.68	87.17333	88.56	90.61333
2	88.29333	89.06667	85.09333	90.74667	90.74667
3	82.53333	82.98667	86.72	88.26667	95.09333
4	77.44	87.81333	84.72	90.53333	90.88
5	77.09333	88.08	91.09333	89.81333	93.57333
Specificity					
1	79.1309	81.5235	86.84917	88.36834	90.55076
2	88.09908	88.94283	84.64419	90.5762	90.5762
3	82.14477	82.65306	86.2955	88.05167	94.9408
4	76.95187	87.53358	84.38003	90.27405	90.73276
5	76.73294	87.72118	91.03672	89.65517	93.61817
FPR					
1	20.8691	18.4765	13.15083	11.63166	9.449244
2	11.90092	11.05717	15.35581	9.423802	9.423802
3	17.85523	17.34694	13.7045	11.94833	5.059203
4	23.04813	12.46642	15.61997	9.725954	9.267241
5	23.26706	12.27882	8.963283	10.34483	6.381828
NPV					
1	79.55771	81.39159	87.27077	88.51133	90.45307
2	88.24164	88.94283	85.32902	90.72276	90.72276
3	82.63215	83.00971	86.94714	88.24164	95.14563
4	77.61597	87.86408	84.78964	90.61489	90.83064
5	77.02265	88.24164	90.93851	89.75189	93.3657
MCC					
1	0.590381	0.633548	0.743457	0.771176	0.812241
2	0.765842	0.781306	0.701878	0.814914	0.814914
3	0.650657	0.659713	0.734409	0.765311	0.901859
4	0.548807	0.756252	0.694386	0.810656	0.81758
5	0.541832	0.761599	0.821843	0.796244	0.87145

At the K-fold value of 1, the accuracy of the implemented RNU-SOA-ADHyNet network is 90.6%, which is 21.05% better than CSA-ADHyNet, 12.7% better than FA-ADHyNet, 5.8% better than NGO-ADHyNet, and 4.8% better than SOA-ADHyNet (as shown in Table 3).

Using Table 6, the accuracy of the proposed RNU-SOA-ADHyNet model is 90.6%, which is 13.9%, 10.9%, 3.9%, and 2.3% better than traditional models like AD-GRU, Res-LSTM, GRU, and DHyNet at a K-fold value of 1. Achieving a substantial improvement in accuracy over existing models suggests that the proposed RNU-SOA-ADHyNet model employs more effective algorithms or techniques that enhance its ability to correctly classify authentication tasks. If the proposed RNU-SOA-ADHyNet model consistently outperforms traditional models in various scenarios, this indicates that this model is more reliable for high-security purposes in vehicular networks.

### Performance evaluation of proposed model comparable with today’s best algorithms

The following Table 8 presents the performance comparison of the implemented MEC security framework in VANET with respect to today’s best algorithms, such as Public Key Infrastructure (PKI) + ECC and the Bidirectional LSTM (BiLSTM) model. These algorithms are validated with respect to measures like fast response speed, real-time performance, algorithmic complexity, computational overhead, and communication overhead.

**Table 8:** Performance evaluation of proposed approach comparable with today’s best algorithms.

	Proposed model (ADHyNet + HECC + MEC)	Traditional model (PKI + ECC) ^35^	Bi-LSTM Model ^36^
Node Authentication Accuracy (%)	98.4	89.3	95.2
End-to-End Latency (ms)	85 ms	65 ms	110 ms
Algorithmic Complexity	O(n·log n + n·d^2^)	O(n log n)	O(n^2^–n^3^)
Model Inference Time (ms)	12 ms (at MEC)	N/A	20–30 ms
Encryption Time per KB (ms)	9 ms (HECC)	4 ms (ECC)	7–12 ms (AES/ECC)
Blockchain Sync Time (avg per block)	140 ms	N/A	~ 180–200 ms
Communication Overhead (% of bandwidth)	28.7%	12.40%	42.50%
Computation Overhead (FLOPs)	2.8 GFLOPs (MEC-distributed)	0.3 GFLOPs	4.5 GFLOPs (vehicle-side)
Scalability (Max nodes supported)	500 + nodes	100–150 nodes	300–400 nodes
Security Level	High (End-to-end + Blockchain + HECC)	Medium	High
Privacy Preservation Score (0–1 scale)	0.94	0.61	0.82
Throughput (Msgs/sec)	2500 +	1200–1500	1800–2200

The accuracy of the node authentication process using our developed RNU-SOA-ADHyNet model is 98.4%, which is greater than other approaches, such as PKI + ECC and the BiLSTM model. The end-to-end latency of our approach is 85 ms, and the model inference time is 12 ms, which demonstrates the real-time effectiveness of the proposed approach.

The analysis on security illustrates that our proposed blockchain framework, with node authentication and data encryption, delivers more effective performance than other recent models. The results in the table also show that the scalability of our approach is higher than the baseline models.

### Statistical significance test of proposed approach based on p-values

Table 9 illustrates the statistical significance test of the implemented secure MEC model in VANET by considering the p-values. Various measures, such as execution time, latency, accuracy, and PDR, are considered for performing the statistical significance analysis. The p-values are evaluated with respect to the observed data and the selected statistical test, which describes the probability of achieving results as extreme as those observed, assuming the null hypothesis is true. The statistical results prove that our model is significant, indicating that the interpretability of the model is higher.

**Table 9:** Statistical significance test of proposed approach based on p-values.

Metrics	Mean	Standard deviation	Unit	p-value	Significance
Accuracy	97.98	0.1	%	0.004	Significant
FNR	1.086	0.07	%	0.012	Significant
CSI	0.963	0.003	Unitless (0–1)	0.018	Significant
MCC	0.947	0.004	Unitless (− 1–1)	0.006	Significant
Precision	98.19	0.15	%	0.009	Significant
Recall	97.8	0.12	%	0.011	Significant
F1-Score	97.99	0.13	%	0.005	Significant
Execution Time	3.25	0.08	Seconds (s)	0.087	Not Significant
PDR	96.12	0.18	%	0.022	Significant
Latency	21.3	1.2	ms	0.03	Significant

### K-fold cross validation of the implemented model using various measures

The k-fold cross-validation of the designed secure MEC framework in VANET, with user authentication and encryption, is validated, and the results are provided in Table 10. Diverse measures, including FNR, precision, accuracy, MCC, and CSI, are considered for validating the developed approach. The results confirm that the average accuracy of the implemented work is 97.98%, proving that the user authentication of vehicles is accurately performed using the proposed RNU-SOA-ADHyNet method. Hence, the security of the VANET environment is maintained very effectively by using this framework.

**Table 10:** K-fold cross validation of proposed model using various measures.

Fold	Accuracy (%)	FNR (%)	CSI	MCC	Precision (%)
Fold 1	97.85	1.12	0.961	0.945	98.1
Fold 2	98.05	1.08	0.964	0.948	98.3
Fold 3	97.92	1.15	0.96	0.944	98
Fold 4	98.12	0.98	0.968	0.952	98.4
Fold 5	97.98	1.1	0.962	0.946	98.15
Average	97.98	1.086	0.963	0.947	98.19

### Attack scenario validation/formal security proof

Table 11 presents the security analysis of the implemented framework and its resistance level against diverse attack types. The defense mechanism of the developed has been described in this table to effectively analyze the security of the VANET system. Diverse attacks such as eavesdropping, DoS, Sybil attack, man-in-the-middle attack, wormhole attack and blackhole attacks have been analyzed to ensure the security of the model. The results confirmed that the security of our proposed model is highly resistant to various attack types.

**Table 11:** Security analysis on proposed model compared to various attack types.

Attack Type	Defense Mechanism	Resistance Level
Sybil Attack	Blockchain-based identity validation and timestamp-based consensus	High
Replay Attack	Nonce and timestamp validation, HECC secure sessions	High
Eavesdropping	HECC encryption ensures that intercepted data remains unintelligible	High
Man-in-the-Middle (MitM)	Encrypted communication with blockchain verification prevents session hijacking	High
Denial of Service (DoS)	MEC filters malicious traffic early; ADHyNet detects abnormal patterns	Moderate to High
Wormhole Attack	Geographical consistency checks and temporal anomaly detection using ADHyNet	Moderate
Blackhole/Grayhole Attack	Deep learning model detects anomalous packet drops and path inconsistencies	High
Impersonation Attack	ECC and blockchain-backed identity authentication resist spoofing attempts	High

### Blockchain overhead analysis

**Fig. 10:**
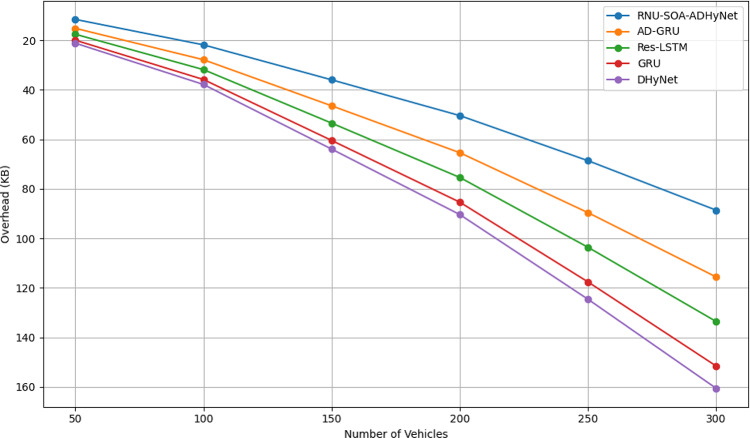
Blockchain overhead analysis

Figure 10 shows the blockchain overhead analysis of the presented ADHyNet with the existing approaches by varying the number of vehicles. When the number of vehicles is taken as 50, the overhead of the suggested ADHyNet model is low. Here, the secure data storage and transmission are leveraged because of the incorporation of blockchain technology, which is more useful for ensuring the distributed ledger across the network. Unlike the conventional approaches, better efficiency is provided by the suggested ADHyNet model as it offers minimum overhead compared to the other approaches.

### Scalability analysis

**Fig. 11:**
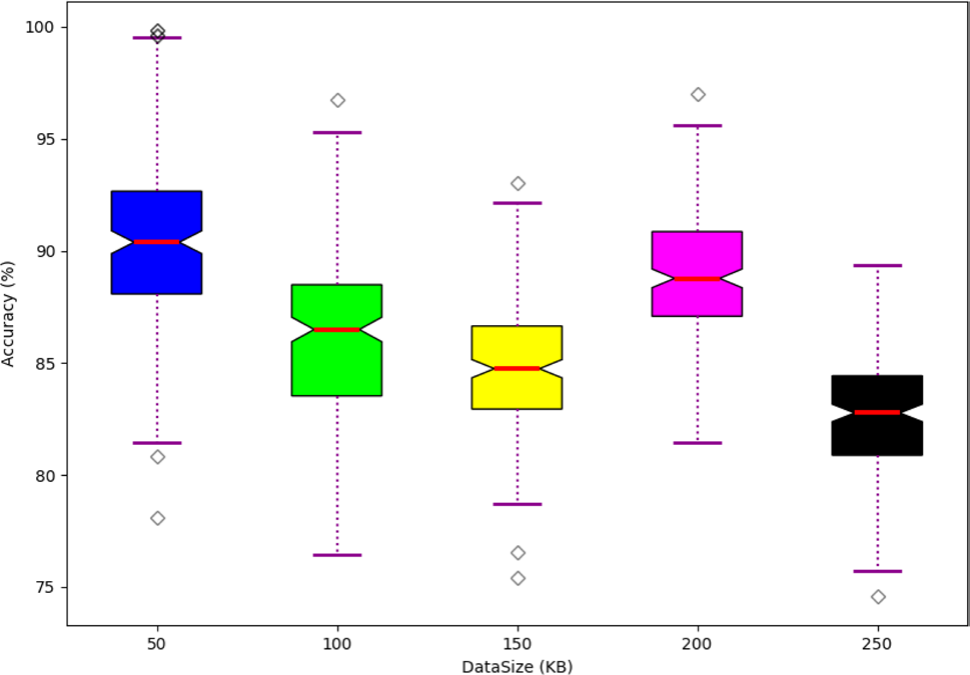
Scalability analysis on developed framework

Scalability analysis carried out in the developed RNU-SOA-ADHyNet-based MEC security framework in VANET is offered in Fig. 11. Here, scalability analysis is performed over the accuracy and data size. Generally, the scalability analysis is carried out to verify the developed model’s efficiency in handling the huge dataset and validation demands while maintaining performance. In this phase, the efficiency of the developed technique is computed over the dataset, and its accuracy is also observed. While analyzing the performance by considering the data size as 50, the developed technique gained a higher performance in the range of 90%. As the count of the data size increases, the performance also lags and falls in the range of 83%. Hence, this validation is good in maintaining the overall efficiency and also reduces the time while training and validating the samples. Thus, the analysis outcomes displayed better outcomes while handling the enormous data in the network.

### Real-time feasibility analysis

Real-time feasibility analysis performed in the designed RNU-SOA-ADHyNet-based MEC security mechanism in VANET is provided in Table 12. In this phase, the efficiency of the developed RNU-SOA-ADHyNet is validated by considering various performance constraints. In the average node speed analysis, the developed RNU-SOA-ADHyNet gained a higher node speed as 29.6 m/s than the prior techniques, which supports better communication in the dynamic conditions. While analyzing the transaction throughput, the developed technique accomplished higher performance and also maintained the reliability by handling heavy traffic scenarios. The efficiency of the developed RNU-SOA-ADHyNet-based MEC security mechanism in VANET is good, and it is also efficient in maintaining the user experiences through smooth services and eliminating the network congestion issues. Thus, the validation outcomes displayed that the suggested mechanism is highly essential to use in a wide range of applications and also effectively enhances the efficiency over different classes.

**Table 12:** Real-time feasibility analysis on proposed RNU-SOA-ADHyNet.

Metric	AD-GRU ^1^	Res-LSTM ^2^	GRU ^32^	DHyNet	RNU-SOA-ADHyNet
Network Size (vehicles)	120	530	1025	5120	9985
Edge/ MEC Nodes	5	18	54	108	210
Average Node Speed (m/s)	10.4	15.7	20.3	25.8	29.6
Communication Range (m)	210.5	255.4	301.8	398.6	497.2
Local Block Time (ms)	48.7	96.4	151.2	199.8	247.5
Global Ledger Anchor Interval (s)	1.2	3.4	5.1	7.2	9.8
Authentication Cache TTL (s)	1.2	3.5	5.6	10.2	28.7
End-to-End Latency (safety msgs, ms)	42.7	63.8	81.4	102.6	118.9
End-to-End Latency (control msgs, ms)	158.4	221.9	297.6	396.2	488.5
Transaction Throughput (tx/s)	524.3	826.5	1043.2	1526.8	1974.6
ADHyNet Inference Time (ms)	5.2	7.6	9.8	12.4	14.9
Packet Delivery Ratio (%)	99.2	98.3	97.1	96.4	95.2
Security Overhead (%)	8.4	10.7	12.2	13.8	14.9
Consensus Nodes per Cluster	3	5	7	9	10
Energy Usage per Vehicle (J/msg)	0.83	1.04	1.27	1.46	1.63
Simulation Area (km^2^)	1	5	10	25	50
Latency Feasibility (%)	99.6	97.9	95.4	92.3	88.5
Blockchain Validation Delay (ms)	52.8	78.9	101.7	124.5	149.3

## Conclusion

A hybrid model was developed in the VANET environment, which integrated node authentication and encryption processes to enhance the reliability and security of the VANET system. This proposed scheme not only achieved high accuracy in communication but also reduced the delay, thereby improving its scalability compared to existing models. By combining adaptive and dilated mechanisms with MEC and blockchain technology, the proposed RNU-SOA-ADHyNet model effectively provides strong, secure node authentication, preserving data integrity and trust through encryption methods.

The RNU-SOA-ADHyNet framework addresses crucial challenges in VANET environments, including unauthorized access and data loss. The proposed model achieved 90.6% accuracy, which is 13.9% better than AD-GRU, 10.9% better than Res-LSTM, 3.9% better than GRU, and 2.3% better than DHyNet. The adaptive features of this proposed model allow it to adjust dynamically to changing network conditions, ensuring optimal performance while protecting sensitive information.

### Research limitations and future scopes

The proposed model needs to address the challenges arising due to response time and performance while numerous vehicles are connected with the network. In some cases, fusing deep learning models with blockchain technology consumes more energy, while limited energy resources are considered in the vehicular network. In order to tackle these issues, a novel energy-efficient technique will be designed by considering the resource management process that supports reducing the overall energy consumption in the network. Moreover, the developed framework needs to tackle the storage issues, while enormous data are processed in the network. In addition, latency issues take place while complex blockchain operations are carried out, which need to be tackled completely to overcome the certain delay that arises in the network. Maintaining the flexibility and reliability in the network is complicated in the dynamic conditions when the vehicles loss their connections to the edge nodes. Additionally, privacy-preserving data-sharing techniques will be explored to further enhance the privacy of sensitive information in different classes.

## Data Availability

The data underlying in this article was taken from the following link: Link:”https://github.com/Djamila-Zamouche/Dataset-of-VANET-communication-traces/tree/main”Access Date: 2025-10-11.
